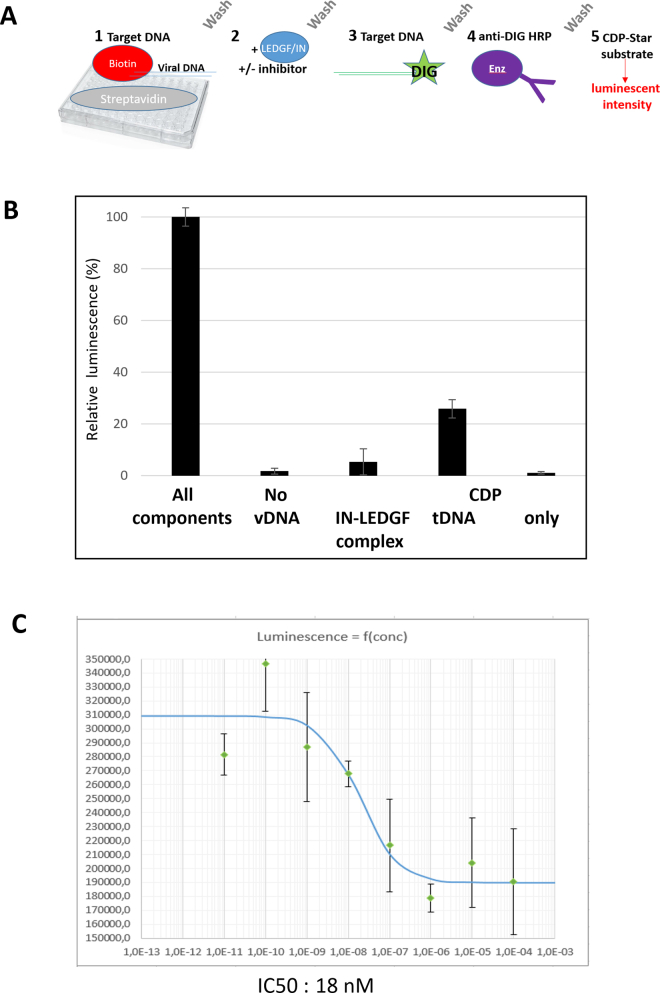# Integrase-LEDGF/p75 complex triggers the formation of biomolecular condensates that modulate HIV-1 integration efficiency *in vitro*

**DOI:** 10.1016/j.jbc.2024.107374

**Published:** 2024-05-16

**Authors:** Claire Batisse, Delphine Lapaillerie, Nicolas Humbert, Eleonore Real, Rui Zhu, Yves Mély, Vincent Parissi, Marc Ruff, Julien Batisse

**Affiliations:** 1 Department of Integrated Structural Biology, Chromatin Stability and DNA Mobility, IGBMC, U-596 INSERM, UMR-7104 CNRS, University of Strasbourg, Illkirch Cedex, France; 2 GDR CNRS 2194 “DYNAVIR” (Viral DNA Integration and Chromatin Dynamics Network), France; 3 Fundamental Microbiology and Pathogenicity Laboratory (MFP), UMR-5234 CNRS-University of Bordeaux, Bordeaux, France; 4 Laboratory of Bioimaging and Pathologies, CNRS UMR 7021, Faculty of Pharmacy, University of Strasbourg, Illkirch Cedex, France

**Keywords:** human immunodeficiency virus, integrase, intrinsically disordered region, macromolecular crowding, viral DNA, integration mechanism regulation, LLPS biomolecular condensates

## Abstract

The pre-integration steps of the HIV-1 viral cycle are some of the most valuable targets of recent therapeutic innovations. HIV-1 integrase (IN) displays multiple functions, thanks to its considerable conformational flexibility. Recently, such flexible proteins have been characterized by their ability to form biomolecular condensates as a result of Liquid-Liquid-Phase-Separation (LLPS), allowing them to evolve in a restricted microenvironment within cells called membrane-less organelles (MLO). The LLPS context constitutes a more physiological approach to study the integration of molecular mechanisms performed by intasomes (complexes containing viral DNA, IN, and its cellular cofactor LEDGF/p75). We investigated here if such complexes can form LLPS *in vitro* and if IN enzymatic activities were affected by this LLPS environment. We observed that the LLPS formed by IN-LEDGF/p75 functional complexes modulate the *in vitro* IN activities. While the 3′-processing of viral DNA ends was drastically reduced inside LLPS, viral DNA strand transfer was strongly enhanced. These two catalytic IN activities appear thus tightly regulated by the environment encountered by intasomes.

Human immunodeficiency virus-1 (HIV-1) is a retrovirus that still infects more than a million people per year, causing more than 630.000 deaths in 2022 through the acquired immunodeficiency syndrome (AIDS). Despite successful antiretroviral therapy (ART), strongly limiting HIV-1 transmission, the persistence of a long-term proviral reservoir frustrates efforts to achieve a cure. Thus, a strong need to understand host–HIV-1 interactions, particularly the mechanisms underlying viral DNA integration into the human genome is crucial to identify therapeutic and potentially curative targets (1, 2).

Integration of HIV-1 genomic DNA is a key step of this virus replication cycle, enabling the stable insertion of the viral genome into host chromatin. Upon integration, the viral genome can establish a long-term latency phase making this a crucial step and a critical target for ART (3). The key factor of the integration process is the viral Integrase protein (IN), a multifunctional protein involved in several steps of the HIV replication cycle (reverse transcription, cytoplasmic migration, nuclear import, chromatin targeting, and integration in the host genome (4, 5, 6)). HIV-1 IN has two distinct enzymatic activities: first the 3′ processing of viral DNA, a maturation step that generates the 3′OH reactive ends by the cleavage of a specific GC dinucleotide, and the second, the strand transfer that consists of a nucleophilic attack of the generated 3′OH on phosphates of the target DNA allowing the integration of the viral DNA into the host DNA genome (for review see (7)). Inhibitions of both of these IN activities (strand transfer and 3′processing) are correlated with a strong decrease of viral replication (1).

To achieve these activities, IN must be intrinsically very flexible, allowing the enzyme to adopt various conformations, to interact with a large number of different protein partners, and, thus, to perform its diverse functions (3′ processing, chromatin targeting; strand transfer), and diverting cellular proteins from their initial functions (8). Through this flexibility, IN presumably adopts a structural conformation specific to the targeted function depending on its associated partners, so that each structure will be linked with a specific function of the protein. In this dynamic context, IN acts as a "platform" protein to form pre-integration complexes (PICs) that will vary in terms of partners depending on the function achieved: 3' processing, chromatin targeting, strand transfer… (9). With several intrinsically disordered regions, IN can interact with numerous viral or cellular nucleic acid or protein partners, thus forming very dynamic macromolecular complexes (4, 10). A major cellular partner of IN is the cellular protein LEDGF/p75 (Lens Epithelium-Derived Growth Factor), a transcription factor that strongly stabilizes IN (11) and enhances chromatin targeting (12). In addition, LEDGF/p75 interacts through its PWWP domain with the H3 histone tail tri-methylated at position 36 (13). LEDGF/p75 has been recently shown to directly modulate the capability of IN to bind chromatin in the context of a chromosome spread (14). The complex IN-LEGDF/p75 is thus of notable interest, appearing as an optimal target for a new class of IN inhibitors called Allosteric inhibitors (ALLINIs), initially designed for disrupting the interaction between IN and LEDGF/p75 (15, 16). These inhibitors strongly stabilize the IN tetramer, impacting not only the integration efficiency but also later steps such as viral maturation (17).

Recently, many proteins with intrinsically disordered regions (IDR) have been found to be involved in the formation of biomolecular condensates (Liquid Liquid Phase Separation - LLPS) allowing them to operate in a restricted microenvironment (for review, see (18, 19, 20)). The understanding of numerous biological systems indeed involves dynamic macromolecular complexes, whose assembly and activity require excluded volume and/or condensed phase environments (18, 21). Viral infection processes have been reported to rely on LLPS formed by either viral components or cellular cofactors (Reviewed in (22, 23, 24, 25)). In the context of HIV infection, the HIV genome has been detected in nuclear MLOs (Membrane Less Organelles; equivalent of “in cell” LLPS) colocalizing with CPSF6, a partner of the p24 Capsid protein, targeting the integration in the dense regions of chromatin (26, 27). These data suggest that pre-integrative steps of viral replication may involve such LLPS formation.

The purpose of this work is to reproduce condensed phase formation *in vitro* so as to better replicate the local concentrations and physiological environment that the complexes of interest encounter within cells. A detailed understanding of the organization and mechanisms of action of these viral protein complexes will provide a better picture of the complexity of the HIV-1 viral cycle. This knowledge will enable the determination of structural data that are currently missing to identify new inhibitors against viral integration. In this study, we showed that each involved partner including CPSF5/6, LEDGF/p75, IN-LEDGF/p75 complexes and functional intasomes (IN-LEDGF/p75 complexed with the viral DNA) were able to form LLPS *in vitro* under precise conditions. We also showed that IN-LEDGF/p75 LLPS were distinct from the CPSF5/6 condensates. We have found that under such conditions, the enzymatic activities of IN are strongly affected, with a drastic inhibition of the 3’ processing activity but a strong enhancement of the strand transfer activity. Altogether, our data strongly suggest that micro-environments such as LLPS may constitute important regulators of the retroviral integration process and its dynamic.

## Results

### IN and LEDGF/p75 contain intrinsically disordered regions (IDR)

**Figure 1:**
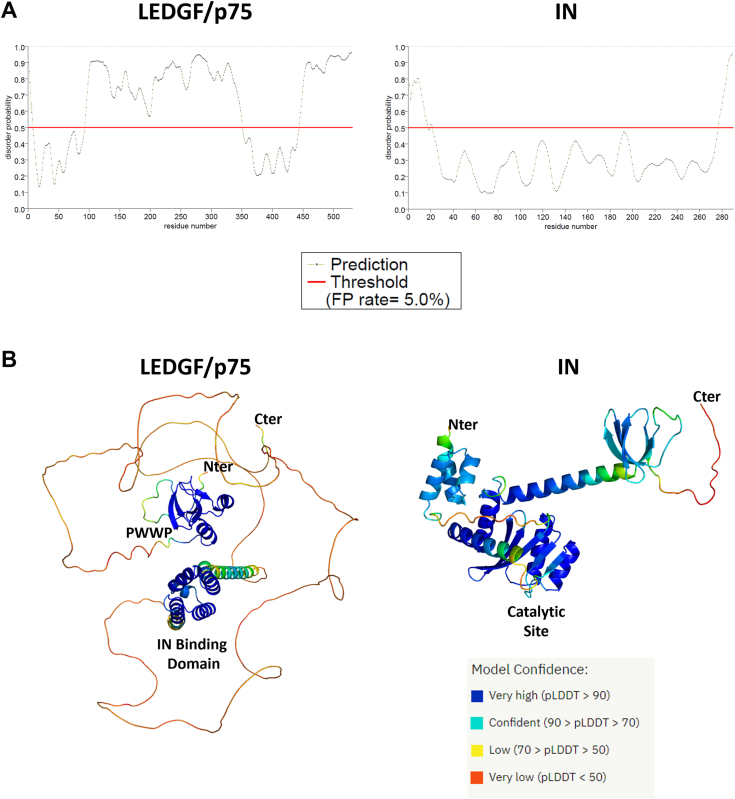
**Both IN and LEDGF/p75 proteins are predicted with several intrinsically disordered regions.***A*, prediction of the disorder probability of LEDGF/p75 (*left*) and IN (*right*) using PrDOS software. Positions above the *red* line were considered as disordered residues, with a standard False Positive (FP) rate set at 5% by the software. *B*, structure prediction (LEDGF/p75 *(left*) and IN (*right*)) using Alphafold2. Color code of the structure according to the confidence (pLDDT: predicted local-distance difference test) are estimated by Alphafold2.

The major common characteristic of LLPS-forming proteins is the presence of intrinsically disordered regions (IDR) (20, 28). To get an overview of the disordered status of both IN and LEDGF/p75, we submitted their amino-acid sequences to the PrDOS algorithm (29) that predicts the disorder of each residue depending on the sequence context (see Experimental procedures). As shown in Figure 1*A*, both IN and LEDGF/p75 harbored IDRs. The IN protein, known to be a protein with high inter-domain flexibility (11), showed disordered domains both at its N- and C-terminal regions. This is confirmed by the structure prediction obtained when the protein sequences were examined using Alphafold2 software (30) (Fig. 1*B*). Contrary to PrDOS prediction, Alphafold2 was able to build an alpha helix structure for the N-terminal region of IN, most likely coming from structures obtained from subdomains of IN (31). The interdomain between the N-Terminal Domain (NTD) and the Catalytic Core Domain (CCD) displays an IDR, as well as at the C-Terminal Domain (CTD) end that appears completely unstructured, confirming the PrDOS prediction. LEDGF/p75 also appeared to be a highly disordered protein, with only two structured domains predicted by Alphafold2: the N-Terminal conserved PWWP motif, involved in DNA binding, notably at the level of mononucleosome (13), and the previously described IN-Binding Domain (IBD) (32). Those predictions are in perfect correlation with a recent review describing proteins involved in biocondensates and impacting the HIV replication cycle (23).

### LEDGF/p75 and IN-LEDGF/p75 complexes are both able to form LLPS *in vitro*

**Figure 2:**
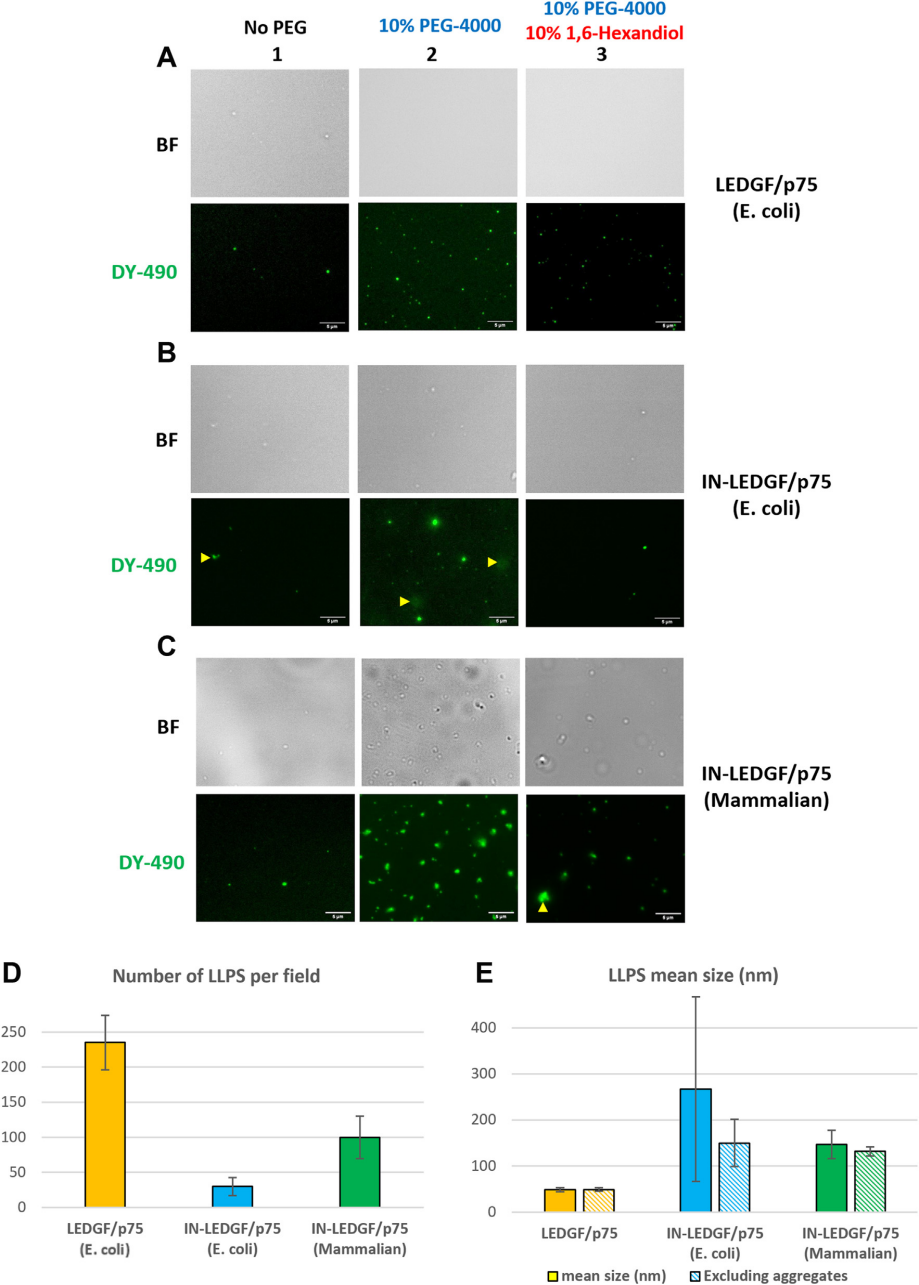
**Both LEDFG/p75 and IN-LEDGF/p75 complexes are able to form LLPS *in vitro*.** Fluorescent LLPS were imaged with a X100 objective (scale bars = 5 μm), using Dye-490 labeled LEDGF/p75 from *E. coli* (*A*), IN-LEDGF/p75 from *E. coli* (*B*) and from mammalian cells (*C*). *Top panels* correspond to brightfield images (BF) whereas *bottom panels* correspond to the fluorescence of the Dye 490. Images were recorded either without LLPS enhancer (column 1) or in presence of 10% PEG-4000 (column 2), or with both 10% PEG-4000 and 10% 1,6-Hexanediol (LLPS inhibitor) (column 3). Yellow triangles point the structures consider as aggregates (not spherical, bigger shape, and not as refringent in BF images). Quantification of LLPS number per field (*D*) and size in nm (*E*). All the analyses were performed using the “Particles Analyzer” module from the ImageJ software. Standard deviations (STD) were calculated from five different images for each condition. LLPS of LEDGF/p75 are depicted in *yellow*, IN-LEDGF/p75 from *E. coli* in *blue*, and IN-LEDGF/p75 from mammalian cells in *green*. Dash bars correspond to the size estimated after exclusion of the biggest particles >1 μm considered as aggregates.

We first investigated if LEDGF/p75 complexes were able to form LLPS *in vitro*. To this end, purified LEDGF/p75 protein was coupled to the fluorescent dye DY490 as described in the Experimental procedures section. LLPS formation was monitored by fluorescence microscopy, in the presence of 10% PEG-4000, a reagent known to favor LLPS (33). As shown in Figure 2*A*, typical spherical condensates were observed in the presence of PEG-4000 (column 2, bottom panel) whereas only rare aggregates were obtained in the absence of this LLPS enhancer (column 1, bottom panel). Condensate formation was found to be inhibited by 1,6 Hexanediol (Fig. 2*A*, column 3), a well-characterized LLPS inhibitor (34). Taken together, these data indicate that LEDGF/p75 forms typical LLPS condensates *in vitro.*

**Supporting Figure S2:**
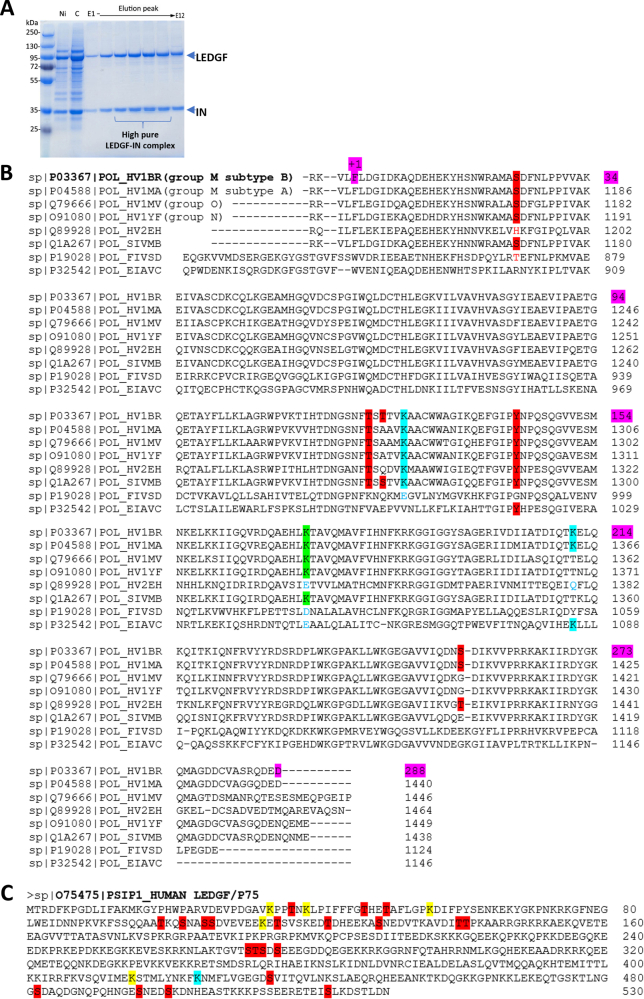


The IN wild-type protein (IN alone) is natively unstable and partially soluble *in vitro*: it needs its interaction with cellular partners like LEDGF/p75 to be efficiently produced and purified (9, 11, 35). Because of this instability, the characterization of LLPS formed with IN alone was problematic due to the systematic presence of aggregates. We nevertheless investigated the capacity of IN alone to form LLPS condensates *in vitro*, in the same experiments done with LEDGF/p75 above. We showed that IN alone, despite a strong tendency to aggregate, was also able to form some LLPS condensates (Fig. S1*A*), less efficiently than the IN-LEDGF/p75 complex (approximately half as much) but in the same conditions (see below). To investigate the LLPS characterization of the IN protein in a more functional environment, we, therefore, decided to use the IN protein in complex with its LEDGF/p75 cellular partner. In addition to being a functional molecular species, IN in complex with LEDGF/p75 has an increased stability and solubility *in vitro*, as we previously described (35, 36), leading to an enhanced enzymatic activity. Thus, we investigated the propensity of IN-LEDGF/p75 complexes to form LLPS *in vitro*. For that purpose, we used two sources of IN-LEDGF/p75, an IN-LEDGF/p75 complex reconstituted from *E.coli* purified IN and LEDGF/p75 proteins (Fig. 2*B*) and a mammalian expressed IN-LEDGF/p75 complex (Fig. 2*C*) produced as a complex in BHK21 cells (see purification gel in Fig. S1*A*), having undergone the post-translational modifications (PTM) that could be crucial for the nucleation of LLPS (37, 38). As expected, both IN-LEDGF/p75 complexes showed an ability to form LLPS in the presence of PEG-4000 *in vitro* (Fig. 2, *B* and *C* column 2), but the mammalian complex was much more efficient resulting in more abundant LLPS, suggesting an important role of PTM in the formation of LLPS condensates, as previously described (39). The presence of PTM were therefore checked by mass spectrometry and numerous modifications such as phosphorylation, acetylation, and methylation were identified all along the two proteins (Fig. S2, *B* and *C*). Most of the modifications on the IN protein were present on conserved residues between the human lentivirus family (Fig. S2*B*), suggesting a conserved role of these PTM. Interestingly, we identified among modified residues several already described, such as serine phosphorylation on S24 and S255 (40) and lysine acetylation K173 (41, 42), both involved in HIV-1 replication efficiency.

The visibility of all LLPS under brightfield light (Fig. 2 upper panels) confirms the formation of *bona fide* condensate phases. Similar to LEDGF/p75 LLPS, the one observed with the IN-LEDGF/p75 complexes was also sensitive to 1,6 Hexanediol (Fig. 2, *B* and *C* column 3).

The size and number of LLPS for each protein or protein complex (Fig. 2 column 2) were quantified as depicted in Figure 2, *D* and *E*. First of all, we observed thrice more LLPS formed in presence of IN-LEDGF/p75 complexes from mammalian cells as compared to the complexes formed in *E. coli* (Fig. 2*D*). Investigating the size of these LLPS (Fig. 2*E*), the complex from *E. coli* appeared to form bigger particles (indicated by yellow triangles in Fig. 2*B* column 2) that we consider as aggregates (not spherical, bigger shape (>1 μm), and not as refringent in brightfield images). Such large structures were only detected with the IN-LEDGF/p75 complex from *E. coli* (Fig. 2*E*), and their high diversity in size (showed by the high standard deviation (STD) values) strengthened our former hypothesis of aggregates rather than condensate LLPS. Interestingly, when we excluded these aggregates (>1 μm) from the size analysis (Fig. 2*E*, blue dash bars), STD was drastically reduced and the LLPS size was close to the one estimated for the mammalian IN-LEDGF/p75 complexes. Since no significant aggregates were detected with the latter complex, their exclusion from the size analysis did not change its size estimate (Fig. 2*E*, green dash bars). Concerning LEDGF/p75 LLPS, image quantifications showed that LEDGF/p75 from *E. coli* was able to form twice as many LLPS condensates than IN-LEDGF/p75 mammalian complexes (Fig. 2*D*) but these LLPS were smaller in size (Fig. 2 column 2 and 2*E*), highlighting a specific molecular organization of LLPS according to their protein composition.

**Figure 3:**
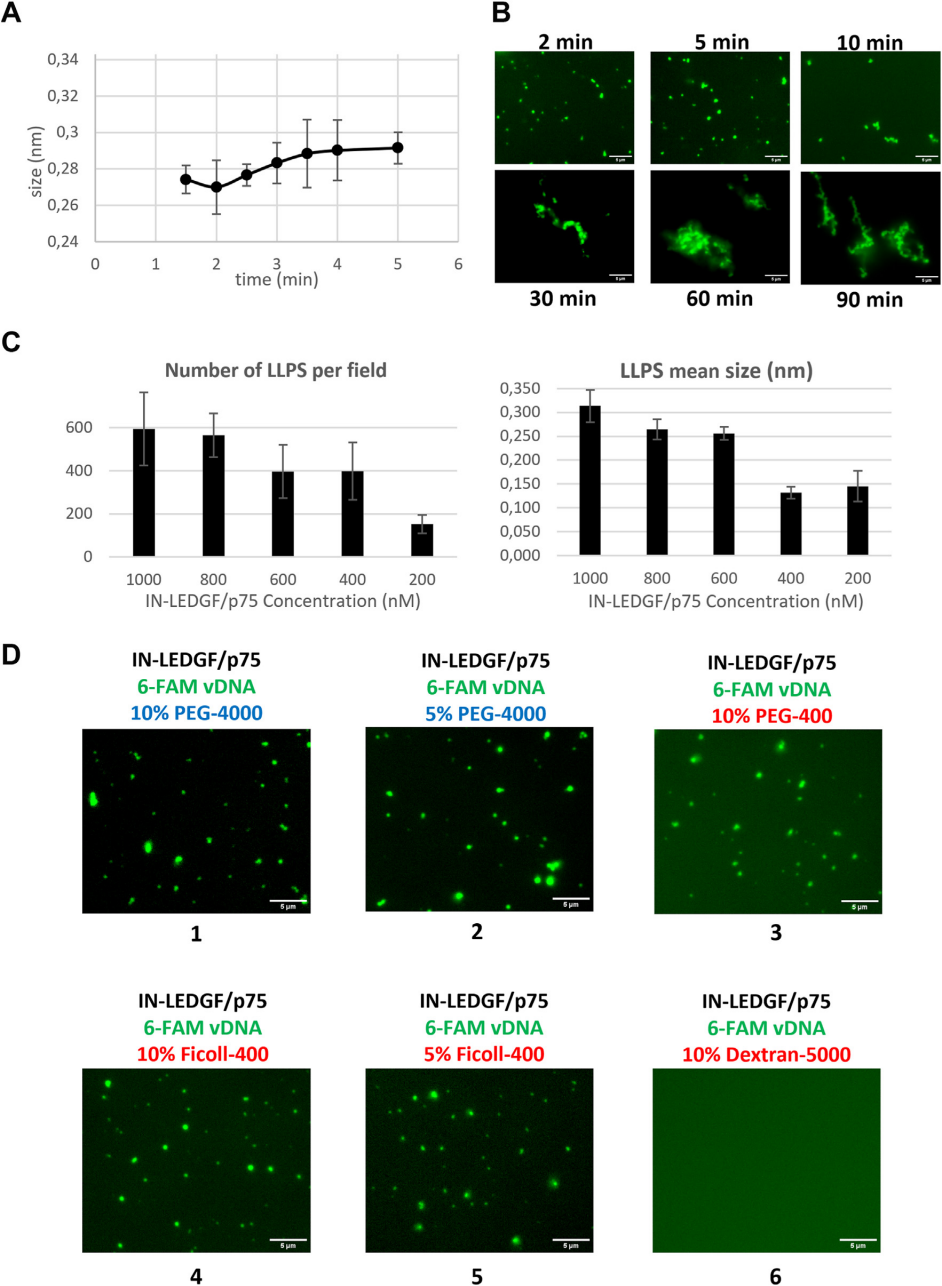
**Characterization of IN-LEDGF/p75 complex LLPS.***A*, size evolution of LLPS size plotted *versus* time in min. Size estimation was performed using the “Particles Analyzer” module from the ImageJ software. Standard deviations (STD) were calculated from five different images for each condition. *B*, fluorescent LLPS were imaged from longer kinetics (indicated time after mixing with LLPS reagent) with an X100 objective (scale bars = 5 μm), using Dye-490 labeled IN-LEDGF/p75 complex from mammalian cells. *C*, quantification of LLPS number per field (*left panel*) and size in nm (*right panel*) depending on the concentration of IN-LEDGF/p75 complex. All the analyses were performed using the “Particles Analyzer” module from the ImageJ software. Standard deviations (STD) were calculated from five different images for each condition. *D*, fluorescent LLPS were imaged using other LLPS reagents with an X100 objective, using Dye-490 labeled IN-LEDGF/p75 complex from mammalian cells. Scale bar = 5 μm.

To further characterize these LLPS, we investigated their behavior over time, concentration, and depending on the reagent used to nucleate them. As shown in Figure 3*A*, we observed a small increase in size over time but rapidly the size reached a plateau around 0.3 μm. Analyzing longer time, we didn’t see any further fusion events that would lead to an increasing size of the LLPS but more an accumulation of LLPS, forming worm-like chains (Fig. 3*B*) starting to appear after 10 to 30 min. Such structures, still seen after 90 min, were already described for other LLPS droplets formed *in vitro* with increasing amounts of BSA protein (43).

When we decreased the concentration of IN-LEDGF/p75 complex, from 1 mM to 200 nM, we still were able to see LLPS, but both their size and more notably their number decrease (Fig. 3*C*) showing a clear effect of the concentration of proteins on their ability to form LLPS. Nevertheless, it should be noted that LLPS were still observed at a 200 nM concentration, closer to the physiological one that could be reached inside the cell nucleus (compared to the 0.8 mM used in our previous experiments).

We finally wanted to validate that our LLPS observations were not restricted to the use of 10% PEG-4000. We therefore investigated the LLPS formation using a lower amount of this reagent. The LLPS were still observed by reducing the percentage of PEG-4000 used to five or 2% (Fig. 3*D*, panel one and 2). We also tested smaller PEG molecules such as PEG-400 that still allowed the LLPS observation (Fig. 3*D*, panel 3). When we changed the nature of the reagent used to nucleate the LLPS, using 10 or 5% Ficoll-400 (44), LLPS were also observed (Fig. 3*D*, panels four and 5) showing that these LLPS were not specific for the PEG environment. On the contrary, the use of 10% Dextran-5000 (another LLPS enhancing reagent (45)) instead of 10% PEG-4000, did not allow the formation of LLPS in our buffers (panel 3) indicating that LLPS were not systematically observed with the IN-LEDGF/p75 complex but required a specific environment provided both by PEG or Ficoll reagents.

Altogether, these results showed that both LEDGF/p75 proteins and IN-LEDGF/p75 complexes are able to form *bona fide* LLPS *in vitro* in the presence of 10% of PEG-4000 or Ficoll-400, visible under brightfield light and sensitive to 1,6 Hexanediol. Since the mammalian IN-LEDGF/p75 complex forms LLPS much more efficiently than the *E. coli* one, we decided to focus our study on the mammalian complex for the next experiments.

### Intasomes (IN-LEDGF/p75 + DNA) also form LLPS *in vitro*

**Figure 4:**
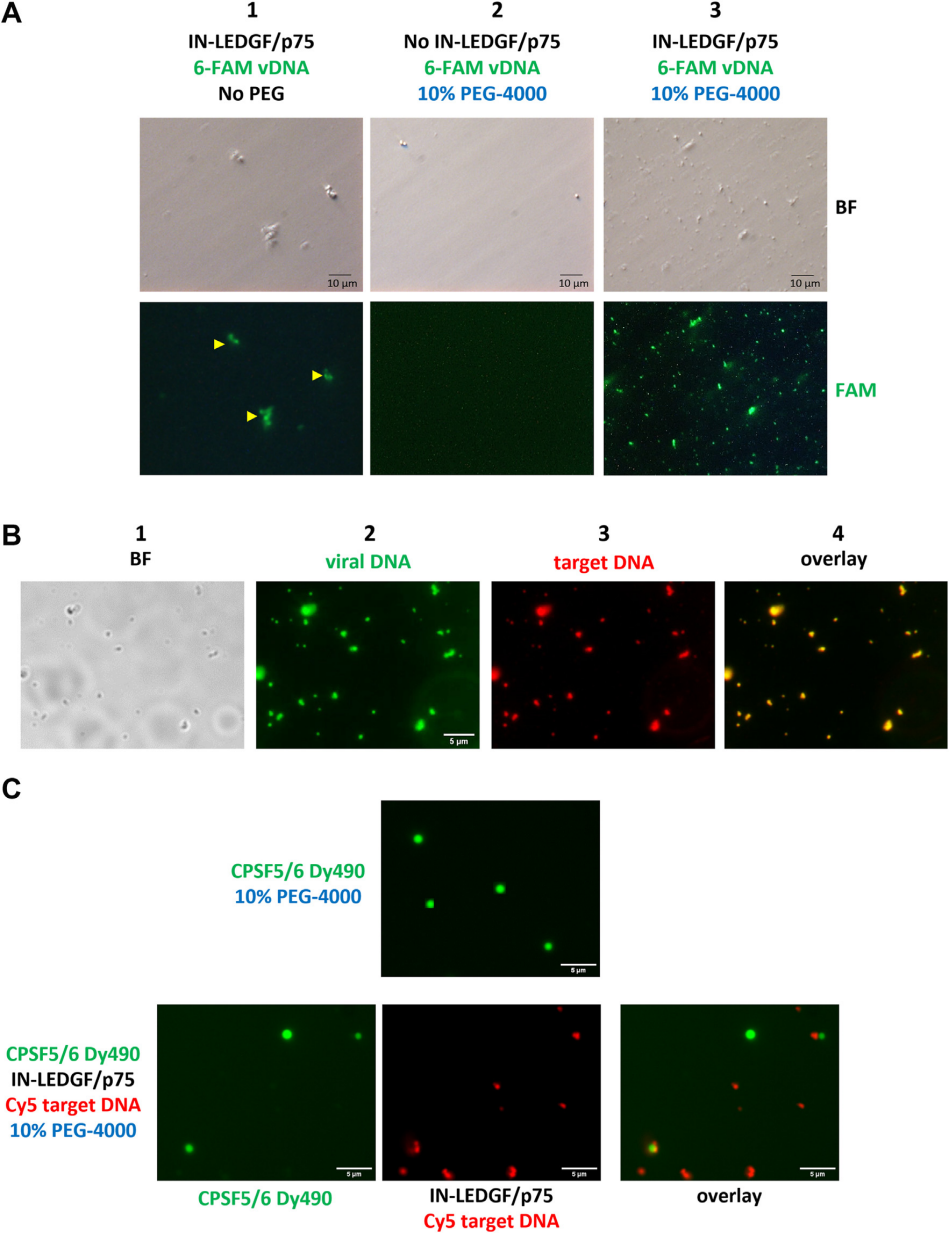
**Intasomes constituted with IN-LEDGF/p75 and DNA form LLPS *in vitro*.***A*, fluorescent LLPS were imaged with a X100 objective, in presence of viral DNA labeled in 5′ with the 6FAM fluorophore. Column 1 is without PEG-4000; column 2 is a control without protein, column 3 is the condition with 10% PEG-4000 and the protein. *Top panels* correspond to the brightfield images (BF) whereas *bottom panels* correspond to the fluorescence of the 6-FAM Dye (scale bars = 10 μm). *B*, fluorescent LLPS were monitored in the presence of the 2 DNA molecules forming the complete intasomes: one viral DNA (6-FAM labeled) and one target DNA (Cy5 labeled). Column 1: brightfield image, column 2: 6-FAM signal (viral DNA), column 3: Cy5 signal (target DNA) and column 4: overlay (scale bars = 5 μm). *Yellow triangles* point to the structures consider as aggregates (not spherical, bigger shape, and not as refringent in BF images). *C*, LLPS were imaged with an X100 objective, with 10% PEG-4000, using Dye-490 labeled CPSF5/6 complex alone (*top panel*) and with IN-LEDGF/p75 complex in presence of target DNA labeled in 5′ with the Cy5 fluorophore (*bottom panel*). Column 1 corresponds to the CPSF5/6 fluorescent signal (Dye-490), Column 2 to the IN-LEDGF/p75 complex fluorescent signal (Cy5 target DNA) and Column 3 is the overlay.

We next investigated the behavior of functionally reconstituted IN-LEDGF/p75 intasomes in this LLPS context. For this purpose, we used an unlabeled IN-LEDGF/p75 complex, pre-mixed with a DNA harboring the specific U5 sequence, mimicking the viral DNA (vDNA) extremity, labeled with 6-FAM at the 5′ end. The reconstruction of intasomes was performed in the same buffer as in Figure 2. LLPS were visualized by fluorescence microscopy (Fig. 4*A*).

We followed the fluorescence in the absence or in the presence of 10% PEG-4000. In the absence of PEG-4000 (Fig. 4*A*, column 1), we were unable to see any condensate phase, but only some aggregation events (indicated by yellow triangles; not spherical, bigger size and not as refringent on brightfield images, as explained above (Fig. 2)). In presence of 10% PEG-4000, we observed discrete LLPS fluorescent dots (Fig. 4*A* column 3), witnessing the ability of intasomes to form LLPS *in vitro*. Moreover, the LLPS were also visible on brightfield images (Fig. 4*A*, upper panels), confirming the formation of real condensate phases. As a negative control, we checked the absence of LLPS signals in the absence of IN-LEDGF/p75 complexes showing their key role in LLPS formation (Fig. 4*A* column 2). This validates also that fluorescent spots observed came from *bona fide* intasomes and not signals from fluorescent DNA aggregation. We finally checked that such LLPS were visible in the different buffers used afterward in this study for the functional assays (see below).

The next step was to study intasomes containing two molecules of DNA, mimicking the physiologically functional intasomes able to perform integration. For such a purpose, we used a viral DNA labeled with a 6-FAM fluorophore (vDNA), and a second DNA labeled with Cy5 with a random sequence to mimic the target cellular DNA (tDNA). We reconstructed the intasomes in the same buffer as in the previous experiment. In the presence of 10% PEG-4000, both fluorescent signals (6-FAM from vDNA and Cy5 from tDNA) were concentrated into small condensate phases (Fig. 4*B* column 2 and 3 respectively). As for the intasomes obtained with viral DNA only, these LLPS were also visible using the brightfield light of the microscope (Fig. 4*B* column 1). Overlap between the signals of the two fluorophores indicated by the yellow dots (Fig. 4*B* column 4) and a Pearson colocalization coefficient (r) of 0.96 ± 0,03 (1 corresponding to a complete overlap – see Experimental procedures), showed that the two DNA molecules are found in the same LLPS. Although our data cannot distinguish between an intasome containing both viral and target DNA from intasomes colocalized bound to only one DNA molecule in the same LLPS, the ability of such intasomes to perform the strand transfer reaction (see below) argued for the existence of intasomes containing both viral and target DNA molecules. Such intasomes were also favored by the experimental procedure of first adding the target DNA (tDNA) saturating all unspecific binding sites and then the specific viral DNA (vDNA) to limit the assembly of intasomes containing only vDNA.

Recently, the formation of in-cell LLPS (also called MLOs for Membrane Less Organelles) after decapsidation has been documented (26, 27) showing that viral DNA together with IN were indeed found in the nucleus within MLOs also containing CPSF6, an important p24 Capsid protein partner (46). We therefore investigated if IN-LEDGF/p75 colocalized with CPSF6 in the LLPS environment *in vitro.* This protein has been produced in our mammalian expression system (see Experimental procedures) together with its partner CPSF5. The purified proteins were then labeled with 490-Dye as we performed for IN-LEDGF/p75 above (Fig. 2) to visualize it using green fluorescence. As shown in Figure 4*C* (top panel) CPSF5/6 complexes were able to form LLPS in the presence of 10% PEG-4000 as well as with 10% Ficoll-400 (not shown). Interestingly, the number of CPSF5/6 condensates was strongly lower than the IN-LEDGF/p75 one despite a higher protein concentration (2 μM) but CPSF5/6 LLPS were larger in size (1 μm *versus* 0.3 μm), suggesting strong differences between the LLPS formed by the two complexes. IN-LEDGF/p75 complex was then added, together with a DNA labeled with Cy5 to visualize it as described above (Fig. 4*B* column 3). In these conditions, we were able to see the two complex condensates, CPSF5/6 in green and IN-LEDGF/p75 in red (Fig. 4*C* lower panel) on the same picture, but the two complexes were forming distinct LLPS condensates, exclusively, with no colocalization between the two: the Pearson colocalization coefficient (r) was 0.16 ± 0,05 (0 corresponding to no colocalization – see Experimental procedures). Consequently, we showed that CPSF5/6 and IN-LEDGF/p75 condensates were not correlated *in vitro*.

### LLPS environment reduces drastically the 3′ processing activity of IN-LEDGF/p75 complex

The next question was to determine whether the IN-LEDGF/p75 complex was active in conditions where it forms LLPS. We first investigated the 3′ processing activity of IN that leads to the removal of the 3′GT dinucleotide from the viral DNA. To follow this activity, we previously developed an anisotropy assay using a 3′ 6-FAM labeled DNA (9). Briefly, if IN is active, it cleaves the 3′end of the viral DNA, releasing a small fluorescent moiety that leads to a decrease in fluorescence anisotropy.

**Figure 5:**
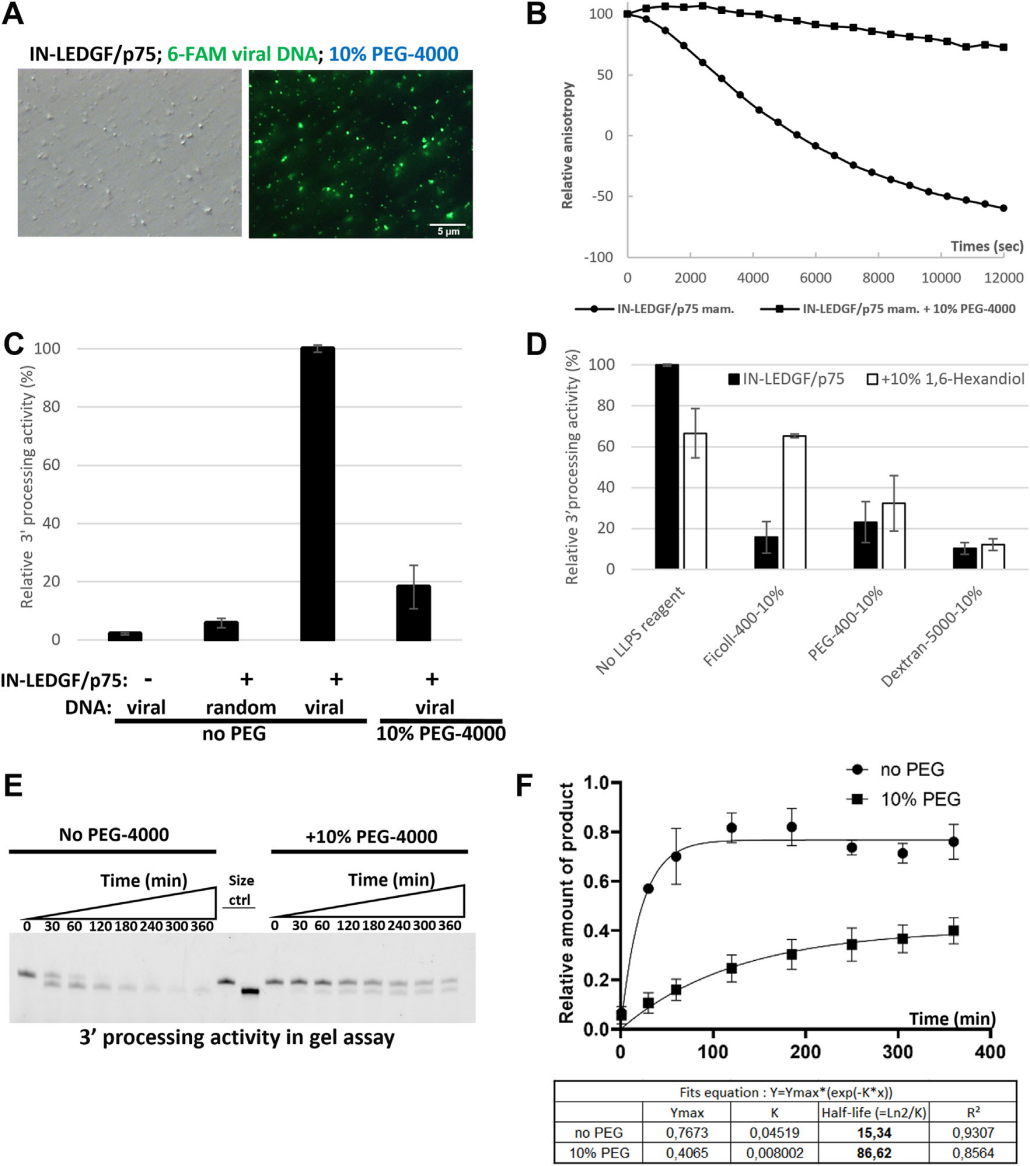
**3′ processing activity is drastically reduced in an LLPS environment.***A*, the IN-LEDGF/p75 complex is able to form LLPS in the 3′ processing reaction buffer (scale bars = 5 μm). *B*, fluorescence anisotropy was recorded at 25 °C in the presence (square) or absence (circle) of 10% PEG-4000. *C*, quantification of the slope observed in *B*, normalized at 100% without PEG-4000. Controls without protein and with an uncleavable random DNA are also shown. *D*, quantification of 3′ processing activity regarding the LLPS reagent used. Anisotropy slope were calculated and normalized at 100% in the condition without PEG-4000. *Black bars* correspond to the IN-LEDGF/p75 complex alone and *white bars* corresponds to the complex in the presence of 1,6 Hexanediol. *E*, gel separation of unprocessed DNA (40 nucleotides DNA, top signal) and processed DNA (38 nucleotides, bottom signal). The *left part* of the gel was performed in the absence of PEG-4000 whereas *right part* was performed using 10% PEG-4000. Kinetics were monitored from 0 to 6 h at 37 °C. *F*, the ratio of product signal to substrate signal was calculated and then plotted *versus* time. *Squares* correspond to data in the presence of PEG-4000 whereas circles describe the data in the absence of PEG-4000. Solid lines correspond to the fits of the data with equations described in the table under the graph. Half-life are in min.

The first question was to see whether the IN-LEDGF/p75 complex together with the viral DNA was able to form LLPS in the conditions of the 3′ processing assay. We therefore repeated the experiment done in Figure 4*A* column 3 in the buffer used for this 3′ processing reaction. As depicted in Figure 5*A*, the IN-LEDGF/p75 complex was able to form LLPS phase condensate in the presence of 10% PEG-4000 in this buffer as efficiently as in the previous buffer, showing that LLPS formation with the IN-LEDGF/p75 complex was not dependent on the buffer used in the previous experiment.

We followed the reaction for 14 h in the absence or in the presence of 10% PEG-4000 inducing LLPS formation (Fig. 5*B*, compare circle *versus* square lines). We found that the IN 3′ processing activity was drastically reduced in the presence of 10% PEG-4000. The value of the slope during the linear phase (first 90 min) was normalized at 100% for the activity measured for IN without PEG-4000 and shown in Figure 5*C*. In the presence of PEG-4000 (*i.e.*, in a LLPS environment), we observed a drastic reduction of the enzymatic activity down to 6% (more than 16-fold decrease). We validated our data using controls without protein and with a random DNA uncleavable by IN.

**Supporting Figure S3:**
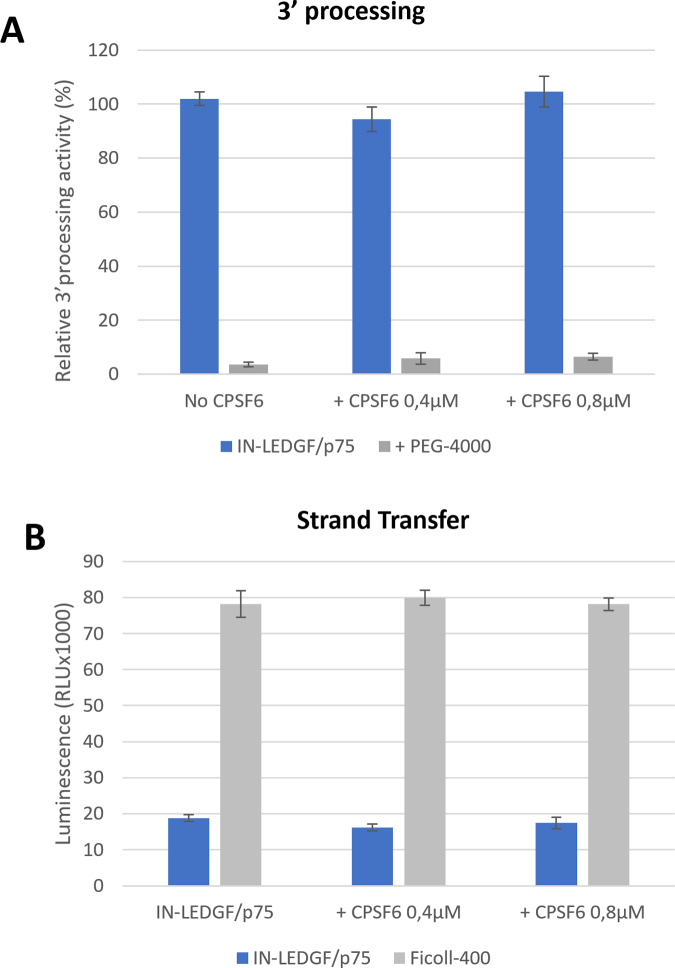


Using the different reagents presented above (Fig. 3*D*), we were able to validate a specific inhibition of the 3′ processing activity of the IN-LEDGF/p75 complex by the LLPS environment regardless the reagent used to nucleate these LLPS. Interestingly, the reversion to a partial activity in the presence of 1,6-Hexanediol particularly seen using Ficoll-400 or PEG-400 comes as an additional argument for the role of the LLPS environment in this inhibition. Note that in the presence of Dextran-5000, that do not lead to LLPS formation, we were not able to measure any activity suggesting a denaturation or disruption of the IN-LEDGF/p75 complex enforced by the non-reversible effect of dextran in the presence of 1,6-Hexanediol. The same experiments were performed using IN alone instead of the IN-LEDGF/p75 complex (Fig. S1*B*), showing the same inhibiting effect of the LLPS environment on the IN 3′ processing activity as for the IN-LEDGF/p75 complex, despite a strongly reduced 3′ processing activity for the IN alone as described before (35). We finally investigated the CPSF5/6 effect on the 3′ processing reaction adding large excess (0.4 or 0.8 μM) of CPSF5/6 in the assay. As shown in Fig. S3*A*, no effect of CPSF5/6 was seen either in the absence or in the presence of the LLPS environment.

To confirm these results, we used another approach based on an in-gel assay using the same DNA but labeled with 6-FAM at its 5′ end. We monitored the kinetics of the 3′ processing reaction, by stopping the reaction at different times with the loading buffer containing urea. Fluorescent signals corresponding to both unprocessed (40 nucleotides) and processed (38 nucleotides) DNA were then separated on a 15% polyacrylamide denaturing gel and visualized using the Typhoon phosphor imager system (Fig. 5*E*). To clearly identify the two different-sized DNAs, we loaded in the middle of the gel (“size control” lines) the 40-nucleotides long unprocessed DNA (labeled with 6-FAM in 5′) and the pre-processed DNA (38-nucleotides long, also 6-FAM labeled in 5′). The kinetics performed in the absence of PEG-4000 (left part of the gel) showed the disappearance of the 40-nucleotide fragment (substrate of the reaction), concomitantly to the appearance of the product (38-nucleotide long DNA). After 1 h, the substrate was already nearly undetectable whereas the product was at is maximal signal. In the presence of PEG-4000 (right part of the gel), the substrate of the reaction never completely disappeared, and the 38-nucleotide product slowly appeared after more than an hour. For quantification, we calculated the ratio (product signal/substrate signal) for each lane (Fig. 5*F*). We found that the appearance of the processed 38-nucleotide product was strongly slowed down in the presence of 10% PEG-4000. The estimated half-time of appearance of the product was approximately 15 min without PEG-4000 and shifted to more than 86 min in the LLPS context. Altogether, these data confirmed that the 3′ processing reaction is drastically impacted in the LLPS environment (10% PEG-4000).

### Strand transfer activity of IN-LEDGF/p75 complex is strongly enhanced by the LLPS environment

The second enzymatic reaction driven by the IN is the strand transfer reaction. To investigate this strand transfer activity (STA), we established a microplate test based on an ELISA protocol. A scheme of this STA assay is depicted in Fig. S4*A*. Briefly, a first biotinylated DNA (viral DNA or vDNA) is loaded on streptavidin-coated microplates. The use of pre-processed vDNA allows to deconvolute the two IN activities and to investigate the STA independently of the 3′ processing activity. Then, the IN-LEDGF/p75 complex is added. After several washing steps, a second DNA (target DNA or tDNA) conjugated to a DIG epitope is added. After a new extensive wash series, the DIG epitope is revealed using an HRP-conjugated antibody that leads to a luminescent product in the presence of an appropriate substrate (see Experimental procedures). We validated our assay with a set of control experiments presented in Fig. S4*B* and checked the behavior of Dolutegravir (S2C), a well-known INSTI inhibitor, that showed an IC50 of 18 nM consistent with previous studies (47).

**Figure 6:**
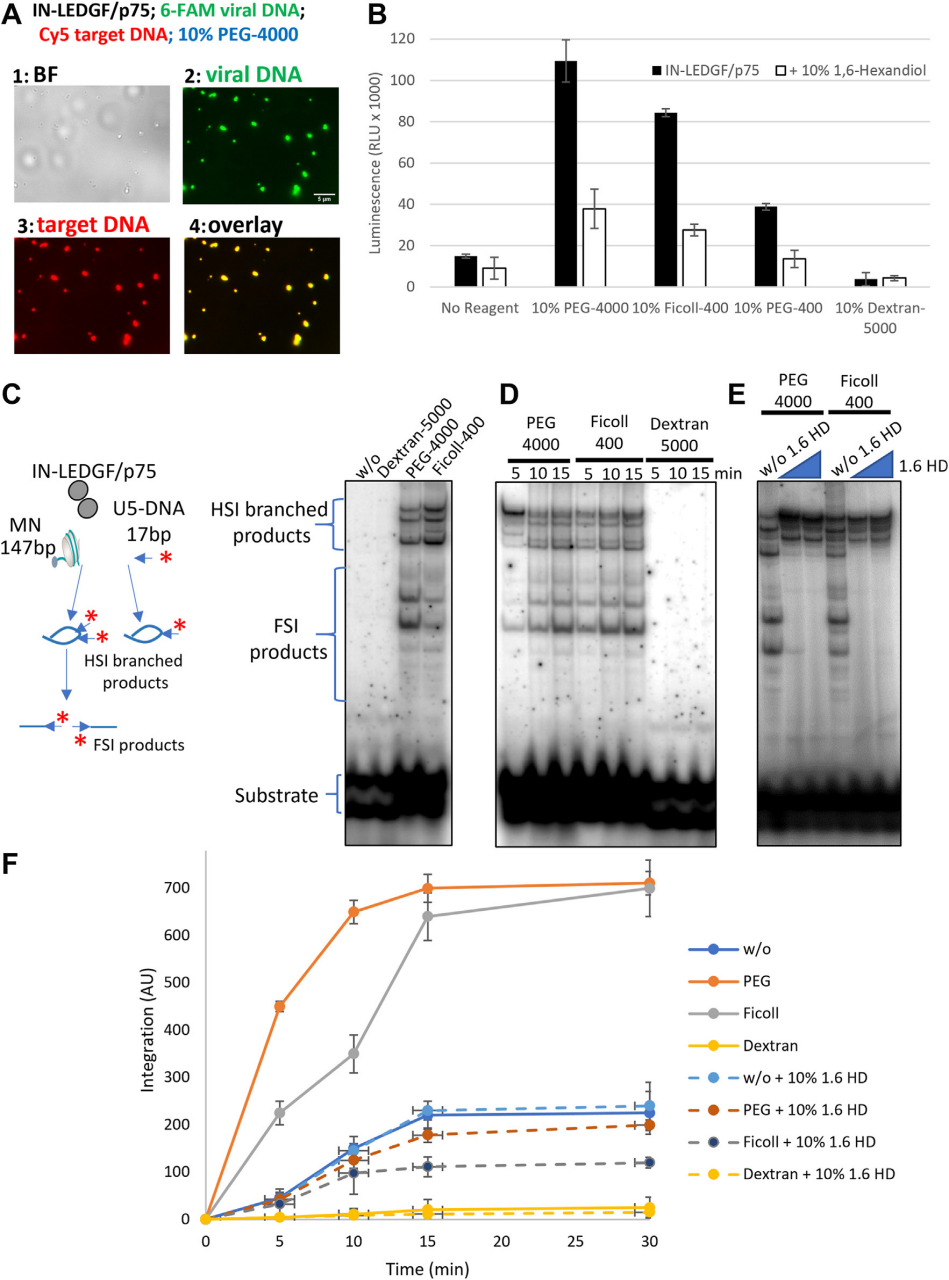
**Strand transfer activity of IN-LEDGF/p75 is strongly enhanced in LLPS environment.***A*, the IN-LEDGF/p75 complex is able to form LLPS in the strand transfer reaction buffer (scale bars = 5 μm). *B*, luminescence signals were measured performing the strand transfer assay either without or with indicated LLPS reagent. *Black bars* correspond to the IN-LEDGF/p75 complex alone and white bars corresponds to the complex in presence of 1,6 Hexanediol. *C*, integration assay using 601 nucleosomes was performed in the presence of either PEG, Ficoll or Dextran as indicated in the Experimental procedures section and products formed after 10 min reaction and deproteinization were loaded onto 8% polyacrylamide native gel. Lane one corresponds to the reaction without IN-LEDGF/p75. *D*, integration assay performed as in *C* and run for 5, 10, and 15 min before monitoring of the integration products after deproteinization and 8% polyacrylamide gel running. *E*, increasing concentrations of 1.6 HD were added to integration reactions run for 10 min before analysis *F*, similar experiments were performed using biotinylated nucleosomes trapped to streptavidin beads, and integration products formed after 10 min of reactions in the different conditions were monitored by quantifying the remaining radioactivity bound to the captured beads Data are reported as mean of at least three independent experiments ± SD. *Blue* = no reagent; *Orange* = + 10% PEG-4000; *Grey* = + 10% Ficoll-400; and *Yellow* = + 10% Dextran-5000. *Dashed lines* correspond to the presence of 10% of 1,6 Hexanediol.

We first checked that the IN-LEDGF/p75 complex together with both viral and target DNA were able to form LLPS in the conditions of the strand transfer assay. We therefore repeated the experiment done in Figure 4*B*, in the buffer used for this strand transfer reaction. As depicted in Figure 6*A*, the IN-LEDGF/p75 complex was able to form LLPS phase condensates in this buffer containing both DNAs as shown by the overlay picture (Fig. 6*A*, panel 4), showing that the LLPS formation of the IN-LEDGF/p75 complex was possible in all buffers we had tested. In terms of activity, as shown in Figure 6*B*, the strand transfer activity was strongly enhanced in the presence of 10% PEG-4000. Indeed, we observed an increase of approximately 7-fold of the luminescent signal in the LLPS environment, showing that such condensate phases can strongly improve the efficiency of the integration process *in vitro*. The same kind of enhancement effect was seen using 10% Ficoll-400 (5-fold enhancement) and a smaller effect (2-fold) was obtained with 10% of PEG-400. As well as for the 3′ processing reaction, no reaction was detected in the presence of 10% Dextran-5000 confirming the denaturation effect of this reagent on our complex. The addition of 1,6-Hexanediol leads to a reverse phenotype confirming the importance of the LLPS environment for this enhancement. The same experiments were performed with IN alone (Fig. S1*C*), showing the same enhancing effect of the LLPS environment on the IN strand transfer activity as for the IN-LEDGF/p75 complex, despite a strongly reduced strand transfer activity for the IN alone compared to the IN-LEDGF/p75 complex.

We also investigated the CPSF5/6 effect both on the 3′ processing and STA reaction adding a large excess (0.4 or 0.8 μM) of CPSF5/6. As shown in Fig. S3*B*, no effect of CPSF5/6 was seen either in the absence or in the presence of the LLPS environment for both IN activities.

To validate our ELISA strand transfer assay and to assess the effect of LLPS on *in vitro* concerted HIV-1 integration onto chromatin, we performed a typical nucleosomal integration assay in conditions promoting, or not, the formation of LLPS. This test allows to detect concerted integration events investigating full-size integration products (FSI) as well as Half-Size Integration products (HSI) (Fig. 6*C*. left panel). As reported in Figure 6*C*, efficient integration onto nucleosome was observed as previously reported (14, 48) only in conditions shown to trigger LLPS *i.e.* using either PEG-4000 or Ficoll-400. In contrast, no integration was observed when Dextran-5000 was used. More accurate monitoring of integration over time showed no difference in the kinetics of the reaction in the PEG-4000 and Ficoll-400 conditions while the use of dextran strongly prevented the formation of integration products (Fig. 6*D*). The addition of an increasing concentration of LLPS inhibitor 1,6 hexanediol leads to a strong blockade of integration in both PEG-4000 and Ficoll-400 conditions (Fig. 6*E*). To confirm these data, we performed deeper kinetics analyses of integration reactions using quantitative integration assay using biotinylated nucleosomes trapped with streptavidin magnetic beads. As reported in Figure 6*E*, data first confirmed the poor integration activity detected in the presence of Dextran-5000 when compared to PEG-4000 and Ficoll-400 conditions. The 1,6-Hexanediol was found to inhibit integration product formation in LLPS-promoting conditions highlighting once more the role of the LLPS condensates in this enhancement effect. By quantifying the enhancement effect of the LLPS environment, the integration efficiency was multiplied by three in the presence of Ficoll-400 and by 3.5 in the presence of PEG-4000, an effect of the same magnitude order as that observed in the ELISA assay. Altogether, the correlation found between integration efficiency (using both of our assays) and LLPS conditions strongly suggests that LLPS promotes efficient *in vitro* nucleosomal integration.

## Discussion

In this study, we have shown first that both the LEDGF/p75 and the IN-LEDGF/p75 complex were able to form LLPS *in vitro*, in the presence of 10% PEG-4000. This is consistent with the predicted characteristics of LEDGF/p75 and IN proteins as flexible proteins with disordered regions, both at the sequence and the structural level (Fig. 1). We showed that LLPS formation was observed in the presence of several LLPS reagents (PEG-4000 – Figs. 2 and 3; Ficoll-400 and PEG-400 – Fig. 3*D*) but not with Dextran-5000 – Fig. 3*D*), confirming as previously described by André *et al.* (45) the dependence of LLPS formation on the reagent used. The LLPS formation also occurs using a smaller amount of reagent (5% *versus* 10%) showing that the LLPS were more sensitive to the nature of the reagents than to its concentration. We also showed that despite a strong tendency to aggregate, IN alone was also able to form LLPS under the same conditions as the IN-LEDGF/p75 complex did, in a less efficient manner, probably due to its low effective concentration (a large amount of IN is sequestered in aggregates).

Interestingly, while the LLPS formed with LEDGF/p75 alone were twice as numerous as the ones observed with the IN-LEDGF/p75 complexes, they were three-fold smaller in size (Fig. 2, *D* and *E*; Fig. 2*A versus* 2, *B* and *C*, column 2), highlighting a specific molecular organization of LLPS according to their protein composition and witnessing the specificity of the LLPS signal observed in each condition. This was confirmed using a third type of LLPS formed by the CPSF5/6 complex (Fig. 4*C*) that also differs from the previous two others. Moreover, the fact that LEDGF/p75 and IN-LEGDF/p75 LLPS differ in number and in size highlights the role of the IN protein in the architecture of these IN-LEGDF/p75 LLPS.

**Figure 7:**
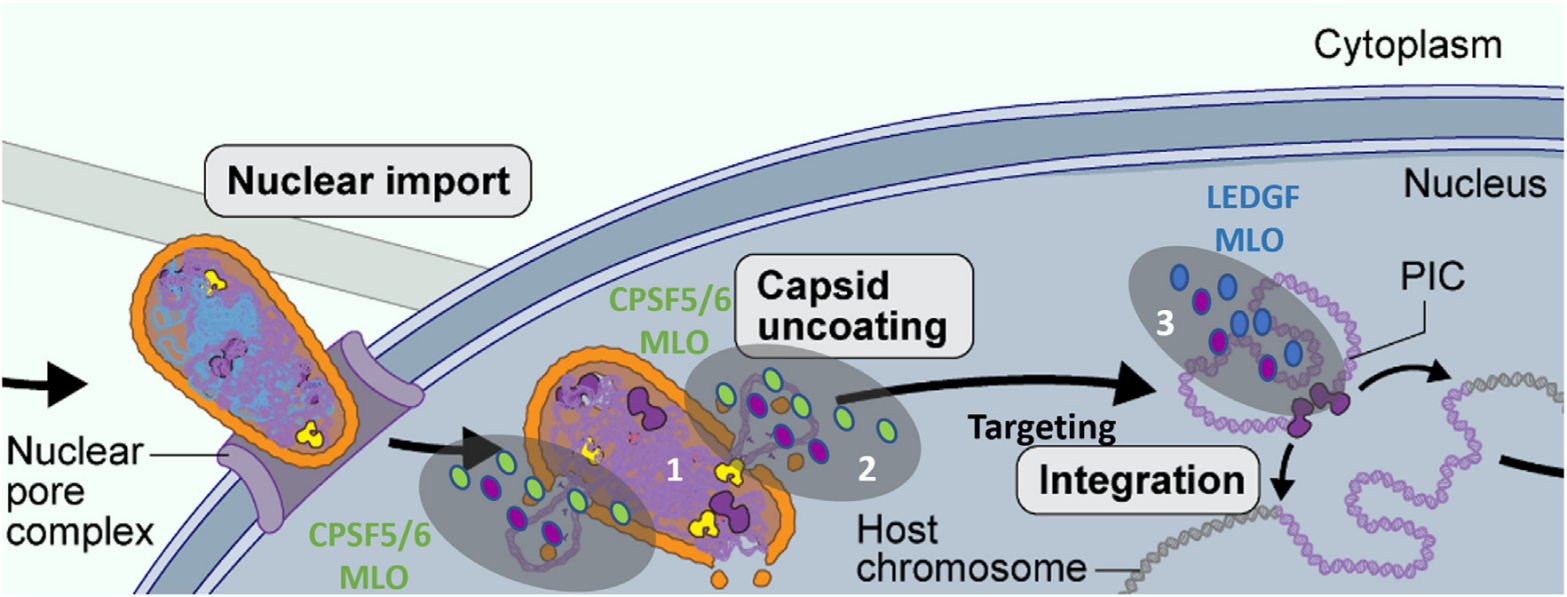
**Dynamic model of pre-integration events emerging from our data.** After the entrance into the nucleus, reverse transcription ended into the capsid (1). Nascent viral 3′ processed DNA together with IN (*purple circles*) is released in the nucleus in a condensates environment allowed by the presence of CPSF5/6 (*green circles*) in the close proximity to the p24 capsid protein (2). The IN-protein recruits then its cofactor LEDGF/p75 (*blue circles*) required for the chromatin targeting, to form the functional intasome that will perform the final integration reaction (3). At this step, the intasome may leave the CPSF5/6 condensates to form its own LLPS environment. Figure adapted from https://scienceofhiv.org/wp/life-cycle/(license: https://creativecommons.org/licenses/by-nc-sa/4.0/).

Interestingly, IN-LEDGF/p75 LLPS were excluded from the CPSF5/6 complex LLPS (Fig. 4*C*) indicating that these two complexes are not part of the same condensates. This observation may explain the absence of the effect of CPSF5/6 on both IN activities *in vitro* (Fig. S3). All of this is in correlation with previous observations from the Di Nunzio group that investigated intracellular CPSF6 MLO (26, 27). The authors showed that IN and viral DNA were found together within CPSF6 condensates but then left those condensates before integration. Combined with our data, this leads us to propose a dynamic view of pre-integration events presented in Figure 7 (1): After the entrance into the nucleus, reverse transcription ended in the capsid (2). Nascent viral 3′ processed DNA together with IN (Fig. 7, purple circles) is released in the nucleus in a condensates environment allowed by the presence of CPSF5/6 (Fig. 7, green circles) in close proximity to the p24 capsid protein (3). The IN-protein recruits then its cofactor LEDGF/p75 (Fig. 7, blue circles) required for the chromatin targeting (12, 14), to form the functional intasome that will perform the final integration reaction. At this step, the intasome may leave the CPSF5/6 condensates to form its own LLPS environment. This model would therefore suggest that the traffic in the nucleus from decapsidation to chromatin targeting/strand transfer could be governed by distinct LLPS environments that may overlap neither in time nor in terms of protein constitution. Further investigations with CPSF5/6 and IN-LEDGF/p75 have to be performed to decipher the *in vivo* time-lapse/relationship between those two complexes and their condensates.

We also showed in this study that the mammalian IN-LEDGF/p75 complexes were much more efficient resulting in more abundant LLPS (3-fold more – Fig. 2*D*), suggesting an important role of PTM in the formation of LLPS condensates, as previously described (39). Mass spectrometry analysis revealed several residues presenting various PTM (phosphorylation, acetylation, and methylation) all along the IN sequence as well as in the LEDGF/p75 one (Fig. S2, *B* and *C*). Concerning LEDGF/p75, it was interesting to note that PTM were mainly observed in the predicted disordered regions, but absent in the regions 280 to 420 which encompass the IN-Binding Domain (347–429) described in the literature (32). Concerning the IN, we found serine phosphorylation on S24 and S255, two residues already described in the literature to be phosphorylated by GCN2 (40) and involved in HIV-1 replication efficiency. The acetylation of the K173 was also previously described and associated with a viral replication impact (41, 42). Interestingly, most of the modified residues inside the IN sequence were residues strongly conserved among human lentiviral integrases (Fig. S2*A*). These conclusions were also in line with our previous work showing that both IN and IN-LEDGF/p75 produced from mammalian cells showed more efficient 3′ processing enzymatic activity *in vitro* (35) suggesting a role of PTM in these activities.

Similar LLPS condensate environments were also observed in the presence of DNA (both viral and target DNA – Fig. 3) indicating that functional intasomes could form LLPS *in vitro*. We therefore decided to investigate and monitor the IN enzymatic activities (*i.e.* 3′ processing and strand transfer) in conditions where LLPS occurred, using optimized functional assays, validated by “in gel” assays. Surprisingly, we showed that the 3′ processing reaction was completely abolished in the presence of LLPS nucleating reagents (Fig. 5, *C* and *D*). The partial recovery of activity observed in the presence of 1,6 Hexanediol (Fig. 5*D*) was an additional argument to attribute the 3′ processing inhibition to the LLPS environment. These results were validated “in gel” to exclude an eventual viscosity effect on the measured anisotropy of some reagents used (Fig. 5*E*). The reaction was much faster when analyzed “in gel” in comparison to the fluorescence anisotropy assay. This may be a consequence of the temperature difference in the two protocols since reactions were performed at 37 °C, whereas fluorescence anisotropy was measured at room temperature (around 20 °C). Such a drastic inhibition of the 3′ processing activity was first astonishing but it validated the crucial role that can play the LLPS environment on the protein conformation. Here, the LLPS environment may block the IN-LEDGF/p75 complex in a specific conformation that does not allow the 3′ processing reaction. We already described such conformation switches mechanism, according to the IN function to achieve in our previous structural characterization of the IN-LEDGF/p75 complex (9).

We further developed an ELISA-based assay to investigate the strand transfer reaction and we showed that in contrast to the 3′ processing activity, the strand transfer reaction was strongly enhanced by the presence of 10% of PEG-4000 and other LLPS reagents Fig. 6*B*), suggesting an important role of the LLPS environment for the integration process. This effect was further validated by “in gel” integration assay (Fig. 6*C*), and the condensate contribution to this strand transfer enhancement was confirmed by the reversion of this effect using the 1,6 Hexanediol LLPS inhibitor. Analyzing the “in gel” integration assay, we saw that the LLPS environment was able to increase the integration events. Moreover, this LLPS environment seems to favor full-size integration products compared to half-size (Fig. 6*D*, compare the top of the gel (Half-size – HSI) and lower part (Full-size – FSI)). This may reflect a functional role of the LLPS environment on the accuracy of the integration reaction to facilitate full integration of the HIV-1 genome into the host genome.

These opposite effects of LLPS on the two integrase enzymatic activities were surprising at first glance but may reflect that the two IN activities were investigated independently *in vitro* whereas *in vivo*, they follow the dynamics of the infection cycle: it’s obvious that in infected cells, these two reactions occur sequentially (the strand transfer reaction required that the 3′ processing activity is achieved) and not in the same environment, neither the same temporality. The timing and the location where the two activities of IN occurred are indeed likely uncoupled during infection (49): the 3′ processing is thought to occur very early in the viral replication cycle in the capsid environment, as soon as reverse transcription occurred (50) whereas the strand transfer takes place at the last step of the early part of the infectious cycle in the nucleus. Recent studies on viral decapsidation have shown that this step occurs later than previously described and that the full capsid enters into the nucleus of infected cells (for review see (51)) implying that the 3′ processing reaction takes place inside the capsid, concomitantly to the end of the reverse transcription (52), before encountering the LLPS environment in the cell nucleus (Fig. 7, from step 1–2). LLPS environments are reencountered by IN after decapsidation in the nucleus (first CPSF5/6 LLPS and then LEDGF/p75 LLPS according to our model (see Fig. 7)). At this very late stage, these IN-LEDGF/p75 LLPS have to be competent to achieve the strand transfer reaction, explaining that the LLPS environment strongly enhanced the strand transfer activity. This also means that inside those LLPS, the IN protein adopts a specific structure optimized for the strand transfer activity, that may not be optimal for the 3′ processing one. Such mechanism was already described in the literature (9) showing that the IN tetramer adopts different conformations inside the IN-LEDGF/p75 complex depending on its partners and particularly depending on the DNA present on the complex: the viral DNA in a complex competent for 3′ processing was shown to be differently positioned in comparison to the same viral DNA in presence of the target DNA (*i.e.* in a complex competent for the strand transfer). Such conformational changes were also mentioned in another structural study of the HIV-1 strand transfer complex (53). Consequently, while there is no physiological interest in the 3′ processing reaction being positively regulated by an LLPS environment since it already occurs inside the capsid, the condensate environment is rather responsible for a conformation blockage of the IN-LEDGF/p75 complex which inhibits the 3′ processing reaction. Conversely, the strand transfer reaction takes place in the nucleus with DNA all around and therefore may require a condensate environment to better control where the integration will occur. The ability of the IN-LEDGF/p75 complex to form LLPS may therefore be crucial at the very late stage of the integration process, when intasomes are excluded from CPSF5/6 LLPS to keep them in an environment compatible with an efficient strand transfer reaction.

Altogether, our *in vitro* results suggest that LLPS or MLO containing IN-LEDGF/p75 take place most likely after decapsidation, triggered by CPSF5/6 MLOs, and constrain the IN protein to switch from a conformation competent for the 3′ processing reaction to another conformation induced by LEDGF/p75, optimal for the strand transfer reaction. As mentioned above, we already described such conformational switching (9) suggesting that a given IN function could be performed by a specific IN conformation regulated by the highly dynamic interacting partners engaged together with IN inside the intasome. Targeting such specific conformations of IN or the IN-LEDGF/p75 complex will be a very interesting approach for the design of new HIV integrase inhibitors that could impede the switch from one conformation to another and therefore impact the different IN functions during the HIV-1 replication cycle. Such specific conformations may occur only in an LLPS environment and therefore would be missed in other “standard” *in vitro* assays used for inhibitor design. *In vitro* drug screening as well as resolving structures at high resolution in the LLPS environment might help the design of new conformational inhibitors that may affect the specificity of HIV integration in euchromatin and therefore target the integration of HIV genome to silenced areas where the genome would not be expressed (Block and Lock strategies).

In conclusion, we have shown in this study that IN-LEDGF/p75 complex was able to form LLPS condensates *in vitro* and that this LLPS environment can modulate the activity of the IN-LEDGF/p75 complex leading to a strong 3′ processing inhibition and a strong enhancement of the strand transfer reaction. This important role of such condensate phases should be considered in the future for new inhibitor design strategies. As perspectives of this work, we aim to more deeply characterize the structural organization of intracellular MLOs observed during the HIV-1 replication cycle *via* imaging techniques such as diffractive microscopy (54), cryo-tomography coupled with sub-tomogram averaging (55), or correlative light electronic microscopy (CLEM) (56).

## Experimental procedures

### Prediction of disorder domains and structure

To predict disordered regions, both LEDGF/p75 and IN (NL4-3 strain) sequences (accession number NP_001121689 for LEDGF/p75 and QGU22154.1 for IN) were analyzed with PrDOS software (https://prdos.hgc.jp/cgi-bin/top.cgi (29)) using a 5% rate as a limit of false-positive prediction (FP rate). Output curves indicate the propensity for each amino acid to be in a disordered context above a 0.5 threshold indicated by a red line. For the structure prediction, we used the alphafold2 software (AlphaFold2.ipynb (30)) using default parameters proposed by the software. PDB structures were then colored using the PyMol software according to the model prediction confidence calculated by Alphafold2.

### Protein expression in *E. coli* and purification

*Purification of human LEDGF/p75 and IN expressed in E. coli.* Both 6His-LEDGF/p75 and 6-His-IN proteins were expressed in BL21 star (DE3) bacteria (ThermoFisher – Table 1). Cells were lysed in lysis buffer containing 50 mM Hepes pH 7.5; 1 M NaCl; 7 mM CHAPS; 2 mM MgCl_2_; 2 mM β-Mercapto-ethanol and protease inhibitors. The protein was purified by nickel-affinity chromatography followed by gel filtration on a Superdex 200 column (GE Healthcare) equilibrated with the same buffer.

**Table 1:** Strain, template and oligonucleotides used in this study

Name and description	Source or note
Strains	
BL21 star (DE3) *E. coli* strain	ThermoFisher
BHK21 C13-2P	ATCC – CCL10
Baby hamster kidney cells	
MVA – IN-LEDGF/p75 (Strain of vaccinia virus MVA)	This study and (37, 38)
MVA – CPSF5-CPSF6 (Strain of vaccinia virus MVA)	
Labelled dsDNA	
6-FAM-38-U5 dsDNA (pre-processed U5 DNA)	This study
5′-(6-FAM)-GACTACGGTTCAAGTCAGCGTGTGGAAAATCTCTAGCA-3′	
5′-TGCTAGAGATTTTCCACACGCTGACTTGAACCGTAGTC-3′	
Cy5-40-Random dsDNA	This study
5′-(Cy5)-AGATACGGCACTGTGGGCAATACGAGGTAATGGCAGACAC-3′	
5′-GTGTCTGCCATTACCTCGTATTGCCCACAGTGCCGTATCT-3′	
40-U5GT-FAM	(9)
5′-GACTACGGTTCAAGTCAGCGTGTGGAAAATCTCTAGCAGT-(6-FAM)-3′	
5′-ACTGCTAGAGATTTTCCACACGCTGACTTGAACCGTAGTC-3′	
Oligonucleotides for Strand transfer Assay	
Biotin-vDNA fwd	This study
5′-Biotin-ACCC TT TTAGTCAGTGTGG AAAATCTCTAGCA-3′	
Biotin-vDNA rvs	This study
5′-ACTGCTAGAGATTTTCCACACTGACTAAAAGGGT-3′	
DIG-cDNA fwd	This study
5′-TGACCAAGGGCTAATTCACT-DIG-3′	
DIG-cDNA rvs	This study
5′-DIG-ACTGGTTCCCGATTAAGTGA-3′	
17 bp U5 viral DNA	(14)
5′-GTGGAAAATCTCTAGCA-3′	

*IN-LEDGF/p75 complex from E. coli reconstitution.* IN-LEDGF/p75 complex reconstitution was performed as published before (14): briefly, both partners were mixed in a 1:2 ratio (LEDGF/p75: IN in respect to the stoichiometry of 4 IN for 2 LEDGF/p75 previously published (9, 11) in their purification buffer. Then, first dialysis was performed overnight using a molecular weight cut-off of 6–8 KDa in the following buffer: 50 mM Hepes pH 7.5; 600 mM NaCl; 1.5 mM CHAPS; 2 mM MgCl_2_, 2 mM β-mercapto-ethanol. Then, the dialysis solution was changed to the final buffer: 50 mM Hepes pH 7.5; 500 mM NaCl; 2 mM MgCl_2_, 2 mM β-mercapto-ethanol (2 bathes) for overnight dialysis. Complexes were then concentrated and purified by gel filtration in this final buffer on a Superdex 200 column (GE Healthcare).

### IN-LEDGF/p75 complex, CPSF5/6 complex, and IN WT expression in mammalian cells and purification

*BHK21 overexpression with MVA expression vector.* We used a vaccinia virus strain (Modified Vaccinia Ankara - MVA - Bio safety level 1 - Table 1) allowing an inducible expression of the IN-LEDGF/p75 complex in BHK21 mammalian cells (37, 38). Briefly, the polygene of interest (coding for the two proteins IN and 6His-LEDGF/p75) was integrated by homologous recombination at the HA locus of the MVA viral genome and the recombinant viruses were selected using a resistance marker cassette present on the polygene as described in (37, 38). BHK21 C13-2P suspension cells (Table 1) were grown in Glasgow's modified Eagle’s medium (GMEM; Thermo Fisher) supplemented with Bacto Tryptose Phosphate (1.5 g/L), 10% fetal calf serum and 50 μM gentamycin. For protein production, a 1.8 L preculture of 10^6^ cells/ml was infected with approximately 0.1 PFU/cell of recombinant virus. Two days later, 12 L of uninfected cells (10^6^ cells/ml) were infected with the preculture. 1 mM IPTG was added at the time of cell mixture. Incubation at 37 °C in 5% CO_2_ at 75% hygrometry was continued for another 24 h. Cells were then pelleted at 2000*g* for 20 min, washed in PBS, and pelleted again at 2000*g* for 10 min. Cell pellets were stored at −20 °C until use. The same expression strategy was performed to produce the CPSF5/6 complex and IN WT protein (IN alone).

*IN-LEDGF/p75, IN WT, and CPSF5/6 Complexes purification.* Concerning IN-LEDGF/p75 complex, pellets from 12 L cell cultures were thawed and resuspended in lysis buffer (50 mM Hepes pH 7.5; 300 mM NaCl; 2 mM MgCl_2_; 10 mM Imidazole; 2 mM β-Mercapto-ethanol). Lysis was performed by pulse sonication on ice (5 min with pulses 2 s ON, 2 s OFF). Lysate was then clarified by ultracentrifugation for 1 h at 100.000*g* at 4 °C. After filtration on cellulose filter 5 μm, the sample was loaded on HisTrap Excel 5 ml column (Cytivia). The column was then washed successively with 10; 20; 40 mM imidazole and complexes were eluted with a gradient from 40 to 500 mM of imidazole. Peak fractions at 280 nm were analyzed on SDS-PAGE and fractions containing the complex were pooled together. The sample was then concentrated on Amicon (Cut Off = 100 KDa) and further purified using a HiLoad Superdex 200 PG 16/60 column (Cytivia) equilibrated with GF buffer (50 mM Hepes pH 7.5; 300 mM NaCl; 2 mM MgCl_2_; 2 mM β-Mercapto-ethanol). Fractions containing the purest complex (checked by SDS-PAGE – Fig. S2*A*) were then pooled together, flash-frozen in liquid nitrogen, and stored at −80 °C. IN WT (IN alone) were purified following the same procedure but in the following buffer: 50 mM Hepes pH 7.5; 1 M NaCl; 7 mM CHAPS (Glycon, D99009); 2 mM β-Mercapto-ethanol, as well as the CPSF5/6 complex in its specific buffer: 50 mM Tris-HCl pH 8.8; 1 M NaCl; 1 M urea; 1 M NDSB-2O1 (Millipore, 480005); 1 mM DTT.

### Labeling of LEDGF/p75, CPSF5/6, IN WT and IN-LEDGF/p75 complexes

For protein labeling with DY490 dye (Dyomics GmbH), 85 μl of purified IN-LEDGF/p75 complex solution (4.65 μM) was pH-adjusted to 8.5 to 9.5 by adding 17 μl of NaHCO_3_ solution (84.01 mg/ml). 2.2 μl of 2 mM DY490 solution (Fluoro-Spin 490, emp-BIOTECH) was added to the pH-adjusted protein solution and incubated for 30 min at 20 °C. The labeled protein was purified by Zetadex-25SF beaded dextran chromatography (CentriPure-Mini-DESALT-Z25 (emp-BIOTECH)) and the level of labeling was checked by measurement of the 493 nm absorbance using Nanodrop.

### *In vitro* phase separation assays

*In vitro* phase separation experiments were carried out in LLPS buffer (50 mM Tris-HCl pH 7.4; 150 mM NaCl) or in another buffer used for enzyme activity tests (see below). PEG-4000 (Hampton research, HR2-529) was included as a crowding reagent to 10% (w/v) final concentration. When indicated, 10% 1,6 Hexanediol (Sigma, 240117) was added in order to inhibit the LLPS formation (34). Other crowding reagents used were: Ficoll-400 (Sigma, F8016), PEG-400 (Sigma, 202398) and Dextran-5000 (Sigma, 31404).

IN-LEDGF/p75 complex concentrations were adjusted so that the final salt concentration was 150 mM NaCl in the assay. Frozen aliquots of proteins were thawed and centrifuged at 20.000*g* to clear aggregates. Fluorescent labelled double-stranded DNA (6-FAM-38-U5 dsDNA and/or Cy5-40-random dsDNA - Table 1) was added to the assay at a DNA/protein ratio of 1:5 when specified. Reactions with all the components were assembled in 1.5 ml tubes and immediately spotted between the glass slide and coverslip (Greiner Bio-one), incubated up to 2 min at room temperature and imaged using a Leica DM-400 microscope equipped with a X100 oil objective. Images were analyzed using Fiji/ImageJ 1.53T software (57) and quantifications were done using the ”Particles Analyzer” module. Means were calculated on 5 to 10 images per condition, and SD from the means of three independent experiments. Colocalization was investigated using Pearson’s colocalization coefficient that calculates the codependency of variations across two channels using linear regression, returning the value r: r = 0 corresponds to no interdependency between the two channels, whereas r = 1 corresponds to a complete interdependency.

### 3′ processing activity assay by fluorescence anisotropy

The reaction based on the anisotropy measurements over time (9), was carried out in 96 well-plates in a final volume of 100 μl as previously described. IN-LEDGF/p75 complex was diluted to 50 nM in reaction buffer (25 mM Tris-HCl pH 7.5; 30 mM NaCl; 6.6 mM MgCl_2_ and 5 mM DTT). 10 nM of a double-stranded DNA labeled at the 3′ end with 6-fluorescein (40-U5GT-6-FAM - Table 1) mimicking the U5 end of HIV-1 DNA were added to start the reaction with or without 10% (v/v) PEG-4000. Fluorescence anisotropy measurements were performed at room temperature (ca. 24 °C) in a spectrophotofluorimeter automat (BMG Pherastar), with a polarized excitation wavelength of 470 nm. The reaction was monitored each 10 min for 20 h. The activity of the complex was calculated from the slope describing the anisotropy decrease over time. Data were normalized relatively to the slope obtained in the absence of PEG-4000, set at 100% complex activity.

### 3′ processing activity “in gel” assay

Reactions were performed at 37 °C in 1.5 ml tubes, in a final volume of 200 μl. IN-LEDGF/p75 complex was diluted to 200 nM in reaction buffer (25 mM Tris-HCl pH 7.5; 30 mM NaCl; 6.6 mM MgCl_2_ and 5 mM DTT). 50 nM of a double-stranded DNA 3′ labeled with 6-fluorescein (40-U5GT-6-FAM - Table 1) mimicking the U5 end of HIV-1 DNA were added to start the reaction with or without 10% (w/v) PEG-4000. Samples were taken at different time points (0, 30 min, 1 h, 2 h, 3 h, 4 h, 5 h, 6 h), quenched with an equal volume of urea denaturing loading buffer and DNA products were separated on 15% polyacrylamide denaturing gels (1X TBE, 7 M urea). Fluorescent signals were visualized using a Typhoon Phosphor-Imager system using an adapted filter (488 nm). The fractions of processed and unprocessed DNA were quantified using Image-Quant (GE Healthcare). Fitting of data was finally performed using the equation: **Y = Ymax∗(exp(-K∗x))**, where Ymax is the value of the plateau reached over time, and K is the calculated rate constant in min^−1^. From this equation, we were able to calculate the half-life in min for each species (processed 38-mer DNA *versus* unprocessed 40-mer DNA) given by ln2/K.

### Strand transfer assay

The strand transfer assays were performed using an ELISA derivative protocol. For dsDNA preparation, single-stranded DIG- or Biotin-oligos were mixed in 1:1 M ratio in annealing buffer (25 mM Tris-HCl pH 7.5; 30 mM NaCl; 0.2 mM MgCl_2_), incubated for 10 min at 95 °C and slowly cooled down to 4 °C. The assays were performed in 96 well plates coated with Streptavidin (Pierce). After a blocking step with TBS, 0.05% Tween-20 and 1% BSA, 100 nM of a first biotinylated dsDNA (Biotin vDNA corresponding to a pre-processed DNA - Table 1) diluted in binding buffer (20 mM Tris-HCl pH 8.0; 1 mM EDTA; 1 M NaCl; 0.2 mg/ml BSA) were bound to the microplate for 1 h at room temperature. After two washes with binding buffer and one with reaction buffer (20 mM Tris-HCl pH 7.5; 15 mM MgCl_2_; 0.1 mg/ml BSA; 5% Glycerol; 5 mM DTT), IN-LEDGF/p75 complex was incubated (50 nM, diluted in reaction buffer). After 1 h at 37 °C and three washes with reaction buffer, 80 nM of a DIG-conjugated dsDNA (Dig cDNA - Table 1) were used to start the strand transfer reaction performed for 1 h at 37 °C with or without 10% (w/v) PEG-4000. Microplates were then washed several times with TBS, 0.05% Tween-20. The Anti-DIG-AP FAP fragment (Roche) was then added at 1/10.000 in TBS; 0.05% Tween-20; 0.1% BSA and incubated for 1 h at room temperature. After five washes, the detection reagent (Tropix CDP-Star Ready-to-use with Emerald II (Applied Biosystem)) was used to reveal DIG binding and after 30 min at room temperature, luminescence was read using a 96 well-plate reader (BMG Pherastar).

### *In vitro* concerted integration assay

Biotinylated native 601 mono-nucleosomes (MN) were assembled as previously described for chromatin assembly (48). Briefly, 5 μg of biotinylated 147 bp Widom fragment (TEBU-bio) were mixed with an excess of 10 μg of human native recombinant octamers produced in *E. coli* (purchased from the “Histone Source” Protein Expression and Purification (PEP) facility from the Colorado State University, https://histonesource-colostate.nbsstore.net) in Tris-HCl pH 7.7 and 2 M NaCl in 100 μl final volume. Salt dialysis was then performed to decrease the salt concentration to 0 using slide-A-Lyzer MINI dialysis device, 7 k MWCO (Fisher Scientific). Assembly was checked by electro-mobile shift assay (EMSA) on 8% native PAGE stained with SYBR safe and SDS-PAGE stained with instant blue.

*In vitro* HIV-1 concerted integration assays were performed as previously reported (14) using recombinant purified IN-LEDGF/p75 complex (200 nM) and biotinylated native 601 mono-nucleosome. Under typical conditions, IN-LEDGF/p75/viral DNA complexes were pre-assembled 20 min at 4 °C in the presence of the 17 bp U5 viral ends DNA hybrid radiolabeled in 5’ (10 nM) and IN-LEDGF/p75 complex. The pre-assembled complex was then incubated with MN (50 ng DNA) for 5 mn in 20 mM HEPES pH7, 10 mM MgCl_2_, 20 μM ZnCl_2_, 100 mM NaCl, 10 mM DTT final concentrations. After the reaction products are treated with proteinase K and phenol/chloroform/isoamyl alcohol (25/24/1 v/v/v) before salt precipitation. Reaction samples are then loaded on 8% native polyacrylamide gel and run for 5 h at 200 V. The gel was then dried and submitted to autoradiography before analysis using ImageJ software. For quantitative assay, the acceptor MN substrates were immobilized on streptavidin-coupled beads before reaction and the reaction products were deproteinized as described above and the integration was quantified by counting the remaining radioactivity bound to magnetized beads. For testing LLPS conditions, 10% PEG-4000, 10% Ficoll-400 or 10% Dextran-5000 were added, and 10% of 1,6 Hexanediol when indicated.

## Data availability

All data are contained within the manuscript. Raw data corresponding to mass spectrometry analysis are found in the File S5 described in the Supporting information section below.

## Supporting information

This article contains supporting information.

## Conflict of interest

The authors declare that they have no conflicts of interest with the contents of this article.

## Figures and Tables

**Figure:**
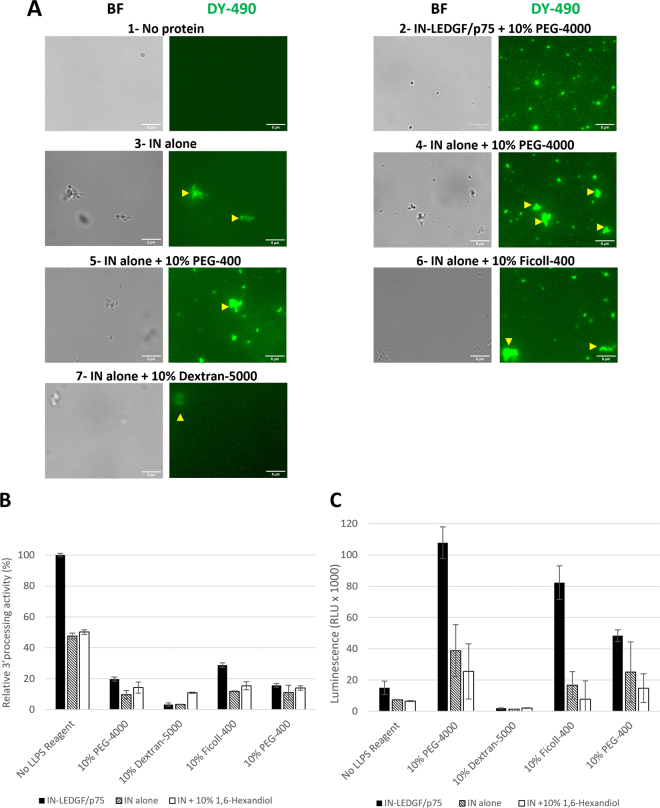
Supporting Figure S1

**Supporting Figure S4:**