# Supplementary material for: Integrase-LEDGF/p75 complex triggers the formation of biomolecular condensates that modulate HIV-1 integration efficiency in vitro
Source: J Biol Chem. 2024 May 16;300(6):107374. doi: 10.1016/j.jbc.2024.107374 (PMC11208922; doi:10.1016/j.jbc.2024.107374)
Supplement: Supplementary file 1 — Supporting Information [file mmc1.docx]

**Supporting INFORMATIONS**

- **Supporting METHODS**

**STA IC50 calculations**: the STA activity measurements were plotted for the different concentrations of compound. The resolution of the Hill’s equation using a Four Parameter Logistic Curve Calculator (<https://www.aatbio.com/tools/four-parameter-logistic-4pl-curve-regression-online-calculator>) allows to get the value of the inflection point, as the dose where the curvature of the response line change, also referred as IC50.

**Mass spectrometry PTM analysis**: purified IN-LEDFG/p75 complex from mammalian cells were trypsin digested and the peptides were extracted twice with acetonitrile/water/formic acid-45/45/10-v/v/v followed by a final extraction with acetonitrile / formic acid (FA) - 95/05 - v/v. Extracted peptides were then analyzed using an Ultimate 3000 nano-RSLC (Thermo Scientific) coupled in line with an Orbitrap ELITE (Thermo Scientific). Peptides were separated on a C18 nano-column with a linear gradient of acetonitrile and analyzed with in a Top 20 collision-induced dissociation data-dependent mass spectrometry with an inclusion list. Data were processed by database searching using SequestHT (Thermo Fisher Scientific) with Proteome Discoverer 1.4 software (Thermo Fisher Scientific). See supplemental Excel file S5 for more details.

- **Supporting FIGURE LEGENDS**

**Figure S1:** **IN WT alone formed LLPS *in vitro* similarly to the one formed by IN-LEDGF/p75 complex with the same effect on IN enzymatic activities:** Fluorescent LLPS were imaged with a X100 objective, using Dye-490 labeled IN WT from mammalian cells. Scale bars = 5 µm. **(A):** Left panels correspond to brightfield images (BF) whereas right panels correspond to the fluorescence of the Dye 490. Images were recorded either without protein (1), with IN-LEDGF/p75 (positive control – 2), without LLPS enhancer (3), or in presence of 10% PEG-4000 (4), or with the other LLPS reagents also tested for the IN-LEDGF/p75 complex (Fig. 3D): 10% PEG-400 (5), 10% Ficoll-400 (6) and 10% Dextran-5000 (7). Yellow triangles point the structures consider as aggregates (not spherical, bigger shape). **(B):** Quantification of 3’ processing activity regarding the LLPS reagent used. Anisotropy slopes were calculated and normalized at 100% in the condition without PEG-4000. Black bars correspond to the IN-LEDGF/p75 complex, hatched bars to the IN alone and white bars corresponds to the IN alone in presence of 1,6 Hexanediol. **(C):** Quantification of strand transfer activity regarding the LLPS reagent used. Luminescence signals were measured performing the strand transfer assay either without or with indicated LLPS reagent. Black bars correspond to the IN-LEDGF/p75 complex, hatched bars to the IN alone and white bars corresponds to the IN alone in presence of 1,6 Hexanediol.

**Figure S2: Purification and** **PTM analysis from IN-LEDGF complex from mammalian cells: (A)** IN-LEDGF/p75 complex purification: Coomassie staining after IN-LEDGF-/p75 complex purification: Ni: after affinity purification; C: after concentration; E1-E12: elution from size exclusion chromatography. Highlighted fractions showing a pure complex that was flash frozen and stored at -80°C. **(B)** sequences alignment of several lentiviral integrase from HIV-1 group subtype B (1^st^ lane); group subtype A (2^nd^ lane); group N (3^rd^ lane), group O (4^th^ lane), from HIV2 (5^th^ lane), from SIV (6^th^ lane), from FIV (7^th^ lane) and from EIAV (8^th^ lane). **(C)** PTM detected in LEDGF/p75. Phosphorylation site are in red, Acetylation in blue, Methylation in yellow, and Acetylation + Methylation in green.

**Figure S3: Effect of CPSF6 on IN enzymatic activities *in vitro*: (A)** Relative 3’ processing activity was measured by our fluorescence anisotropy assay in absence (left) or in presence of CPSF6 (middle and right; 2 different concentrations) in standard conditions (blue bars) or within the LLPS environment (grey bars). **(B)** Relative STA activity was measured in our ELISA assay in absence (left) or in presence of CPSF6 (middle and right; 2 concentrations) in standard conditions (blue bars) or within the LLPS environment (grey bars).

**Figure S4: Strand Transfer Assay (STA) optimization: (A)** Experimental strategy workflow of the STA. **(B)** Control experiments performed with all components (positive control first bar, set up at 100% of STA activity); without biotinylated viral DNA (vDNA, 2^nd^ bar); without IN-LEDGF/p75 complex (3^rd^ bar) without target DNA (tDNA, 4^th^ bar), and with only the luminescent substrate (CDP only, 5^th^ bar). **(C)** IC50 determination for Dolutegravir where luminescence was plotted versus Dolutegravir concentration.

- **Supporting TABLE 1: Strain, template and oligonucleotides used in this study**
- **Supporting FILE S5: “****Supp File S5_MassSpec PTM analysis.xlsx”:** Excel file with all information concerning the mass spectrometry analysis investigating PTM.
